# Fine Mapping of a Vigor QTL in Chickpea (*Cicer arietinum* L.) Reveals a Potential Role for *Ca4_TIFY4B* in Regulating Leaf and Seed Size

**DOI:** 10.3389/fpls.2022.829566

**Published:** 2022-02-24

**Authors:** Duong T. Nguyen, Julie E. Hayes, John Harris, Tim Sutton

**Affiliations:** ^1^School of Agriculture and Environment and Institute of Agriculture, The University of Western Australia, Crawley, WA, Australia; ^2^South Australian Research and Development Institute, Adelaide, SA, Australia; ^3^School of Agriculture, Food and Wine, University of Adelaide, Urrbrae, SA, Australia

**Keywords:** chickpea (*Cicer arietinum* L.), *Ca4_TIFY4B*, fine mapping, leaf size, seed size regulation, plant vigor, *PEAPOD (PPD1/PPD2)*

## Abstract

Plant vigor is a complex trait for which the underlying molecular control mechanisms remain unclear. Vigorous plants tend to derive from larger seeds and have greater early canopy cover, often with bigger leaves. In this study, we delimited the size of a major vigor quantitative trait locus (QTL) on chickpea chromosome 4–104.4 kb, using recombinant association analysis in 15 different heterogeneous inbred families, derived from a Rupali/Genesis836 recombinant inbred line population. The phenotypic and molecular genetic analysis provided evidence for a role of the gene *Ca4_TIFY4B*, in determining leaf and seed size in chickpea. A non-synonymous single-nucleotide polymorphism (SNP) in the high-vigor parent was located inside the core motif TIFYCG, resulting in a residue change T[I/S]FYCG. Complexes formed by orthologs of Ca4_TIFY4B (PEAPOD in *Arabidopsis*), Novel Interactor of JAZ (CaNINJA), and other protein partners are reported to act as repressors regulating the transcription of downstream genes that control plant organ size. When tested in a yeast 2-hybrid (Y2H) assay, this residue change suppressed the interaction between Ca4_TIFY4B and CaNINJA. This is the first report of a naturally occurring variant of the TIFY family in plants. A robust gene-derived molecular marker is available for selection in chickpea for seed and plant organ size, i.e., key component traits of vigor.

## Highlights

The transcriptional regulator *Ca4_TIFY4B*, which is found at the *Ca4_Vqtl* region in chickpea, is associated with the control of vigor-related traits including leaf and seed size.

## Introduction

Chickpea is currently ranked as the second largest pulse produced worldwide after dry beans (FAOSTAT, 2019). Although widely recognized as a major grain legume, chickpea is still mainly cultivated in marginal areas that often experience a range of biotic and abiotic stresses including disease and weed competition, heat, drought, low temperatures, and salt contamination. Increasing pressure has been put on breeders to develop high-yielding varieties that are not only resistant/tolerant to stress but also resilient in the face of climate change. In cool, short-season environments, increasing vigor is a key priority of breeding programs to improve competitive ability during crop establishment and yield. Genetic studies in many different crops have mapped vigor-related loci and assessed their impact under a range of growing conditions including irrigated, drought, salt, and disease pressure (Collins et al., 1999; Graham et al., 2011; Platten et al., 2013; Sivasakthi et al., 2018; Taylor et al., 2019; Atieno et al., 2021; Nguyen et al., 2021). In chickpea, vigor has been reported to contribute either positively (Subbarao et al., 1995; Turner et al., 2001) or negatively (Zaman-Allah et al., 2011) to yield. This suggests that to maximize productivity, genetic tools are needed to enable the selection of a specific vigor profile that is matched to a target environment.

Recently, quantitative trait locus (QTL) mapping and association studies across multiple environments identified major genetic regions for vigor-related traits on chromosomes (Ca) 1, 3, and 4 in chickpea (Nguyen et al., 2021). The vigor QTL on Ca4 (*Ca4_Vqtl*) overlapped with a “hotspot” region of QTL previously reported for drought tolerance (Varshney et al., 2014; Jaganathan et al., 2015; Kale et al., 2015; Singh et al., 2016) and vigor (Sivasakthi et al., 2018). The overlapping QTL region also colocated with a region for seed size in both the ICC4958/ICC1882 (Varshney et al., 2014; Sivasakthi et al., 2018) and Rupali/Genesis836 recombinant inbred line (RIL) mapping populations (Atieno et al., 2021; Nguyen et al., 2021). A positive impact of seed size on vigor has been reported in both cereal and legume species, including wheat (*Triticum aestivum* L.) (Sloane, 1999; Zhao et al., 2019), rice (*Oryza sativa* L.) (Roy et al., 1996), maize (*Zea mays* L.) (Yusuf et al., 2014), barley (*Hordeum vulgare* L.) (Massimi, 2018), birdsfoot trefoil (*Lotus corniculatus* L.) and alfalfa (*Medicago sativa* L.) (Carleton and Cooper, 1972), chickpea (*Cicer arietinum* L.), lupin (*Lupinus angustifolius* L.) and pea (*Pisum sativum* L.) (Kamboozia, 1994), and soybean (*Glycine max* (L.) Merr.) (Fatichin et al., 2013). It is therefore plausible that *Ca4_Vqtl* contains a single causative factor with a pleiotropic effect on both vigor and seed size.

The *Ca4_Vqtl* region is estimated between 12.65 and 13.06 Mb of chromosome 4 (*CDC Frontier Ref V2.6.3*; Edwards, 2016; Ruperao, 2016) and contains 39 genes (Nguyen et al., 2021), making it difficult to prioritize candidates for further investigation and functional confirmation. Further fine mapping was necessary to prioritize candidates with roles in plant vigor. The objective of this study was to improve the resolution of the *Ca4_Vqtl* genomic region and identify candidate genes for vigor-related traits in chickpea. This was achieved using heterogeneous inbred families (HIFs) derived from selfed F_4_ RILs of Rupali/Genesis836, confirmed as heterozygous across the *Ca4_Vqtl* interval. High-depth sequence capture (Nguyen et al., 2021) was used to identify sequence variation. We identified a variant of the gene *Ca4_TIFY4B* in the high-vigor Rupali RIL parent, proposed to alter the regulation of seed size and other plant organs including leaves and roots and contribute to differences in plant vigor.

## Materials and Methods

### Fine Mapping Analysis of *Ca4_Vqtl*

#### Development of Heterogeneous Inbred Families for Fine Mapping

Three F_4_-derived RILs (RIL15, RIL50, and RIL89) from a Rupali/Genesis836 biparental mapping population (Atieno et al., 2021; Nguyen et al., 2021) were identified as heterozygous across the vigor QTL region *Ca4_Vqtl* (12.65–13.06 Mb, *CDC Frontier Ref V2.6.3*). These were selfed to develop fine-mapping families. Sixteen heterozygous F_5_ plants were bulked for seed and are hereafter referred to as F_1_ of the fine-mapping population. F_2_ fine-mapping population seedlings (3,363 individuals) were genotyped with two flanking markers (*Ca4_12652558* and *Ca4_13068179*) to identify recombinants within the *Ca4_Vqtl* interval. Of these, 39 F_2_ recombinants were genotyped with further markers to delineate the recombination breakpoints, and were grown to matuity. Fifteen recombinants, each with at least 50 seeds, were selected as F_2_ HIFs. HIF progenies were genotyped and homozygotes were phenotyped to identify trait-marker associations for each family and to reduce the physical size of the *Ca4_Vqtl* interval.

#### Phenotyping

To investigate the impact of the *Ca4_Vqtl* on vigor-related traits and identify robust component traits for fine mapping, pairs of near-isogenic lines (NILs) contrasting across the *Ca4_Vqtl* region were developed from RIL15, RIL50, RIL89 (F_5_), and RIL161 (F_4_). Single plants (*n* = 6–14) for each NIL were grown in 25-cm diameter pots containing BioGro potting medium^1^ under natural light shade house conditions. At both 6 and 10 weeks after emergence, various measurements were made on each plant. These included Fractional Green Canopy Cover (FGCC) using the Canopeo application (Oklahoma State University, Stillwater, OK, United States),^2^ canopy height, main shoot length, branch length, total stem length, branching index (branch length/main stem length), number of branches, average branch length, leaf number, and internode length. At 13 weeks, FGCC and canopy height measures were again taken, as well as leaf size and petiole length. For leaf size, the fourth, fifth, and sixth youngest fully emerged leaves were excised from the main stem and laid flat on white paper. Images taken with a smartphone were analyzed for green leaf area using the Easy Leaf Area application and a 2 cm × 2 cm red-colored square placed on the same horizontal plane for calibration (Easlon and Bloom, 2014). Days to flowering (DTF) was recorded when observing the first opened flower. At maturity, total biomass (TBM), seed weight (SW), seed number, 100 SW (100SDW), and seed size for each plant were recorded. Seed size was measured as the two-dimensional (2D) area of 20 seeds photographed for each NIL, using the SeedCounter application (Komyshev et al., 2017).

For fine mapping, progenies of 15 HIFs were grown as single plants in pots (19.5 cm height × 14.9 cm diameter) during winter in 2020 (13 HIFs) and 2021 (2 HIFs) in a greenhouse at 20°C with natural lighting. The phenotypic data were collected for leaf size (using fourth, fifth, and sixth youngest fully emerged leaves) at 13 weeks, seed size, 100SDW, and seed number.

An additional phenotyping experiment was conducted in the greenhouse using four HIF-derived NIL pairs. Four plants were grown per pot (17.5 cm height × 19 cm diameter, filled with 3 kg BioGro potting medium), with 3 or 4 replicate pots per genotype. Measures of leaf size (using second and third youngest fully emerged leaves), FGCC, root dry weight, and shoot dry weight were collected at 4 weeks after sowing.

#### Genotyping

Single-nucleotide polymorphisms (SNPs) between Rupali and Genesis836 inside the region of 12.65–13.06 Mb were detected from the sequence capture data that were previously described by Nguyen et al. (2021). SNPs were used to design Kompetitive Allele-Specific PCR (KASP) genotyping assays using the SNPline PCR Genotyping System (LGC, Middlesex, United Kingdom). All materials were additionally genotyped with KASP markers linked to flowering loci on Ca5 (*CaELF3a*; Ridge et al., 2017) and Ca3, and a vigor QTL on Ca3, to ensure these were fixed within each HIF. These loci are described by Atieno et al. (2021) and Nguyen et al. (2021). Primer sequences are detailed in Supplementary File 1.

#### Statistical Analysis

The probability of association between traits and markers for each HIF or NIL pair was investigated using Student’s *t*-tests (*P* < 0.05) (Kalpić et al., 2014). The correlation analysis was performed in Excel.

### Yeast 2-Hybrid Assay

Cloning was conducted using Gateway^®^ Technology (Thermo Fisher Scientific, Waltham, MA, United States). The full-length coding sequences of *Ca4_TIFY4B* (TIFY 4B-like isoform X1) and *CaNINJA* (Novel Interactor of JAZ) were PCR-amplified (for primers, refer to Supplementary File 1) using cDNA isolated from the whole shoot sampled at 2 weeks of Rupali and Genesis836. PCR products were directionally cloned into the entry vector pENTR™/D-TOPO^®^ using One Shot^®^ TOP10 competent *Escherichia coli*. Inserts were confirmed by the sequencing of extracted plasmid DNA from multiple independent clones.

The yeast 2-hybrid (Y2H) analysis using the GAL4-ProQuest™ Two-Hybrid System was performed as described (Myhrstad, 2011; Cuéllar et al., 2013; Erffelinck et al., 2018). Entry vectors carrying the Rupali allele of *Ca4_TIFY4B* (*TIFY4B_R*), the Genesis836 allele of *Ca4_TIFY4B* (*TIFY4B_G*), and *CaNINJA* were site-specifically recombined with two different destination vectors to generate both bait (pDEST32™, containing DNA-binding domain GAL4-DBD) and prey (pDEST22™, containing activation domain GAL4-AD) constructs for each insert. The *Saccharomyces cerevisiae* MaV203 yeast strain, containing GAL4-inducible reporter genes *URA3*, *HIS3*, and *lac*Z, was cotransformed with bait and prey constructs using a polyethylene glycol (PEG)/lithium acetate/single-stranded carrier DNA method (Gietz and Schiestl, 2007). Transformants were selected on synthetic complete (SC) medium lacking leucine (-Leu) and tryptophan (-Trp).

Transformants with bait Ca4_TIFY4B and prey CaNINJA, as well as with bait CaNINJA and prey Ca4_TIFY4B, were characterized separately to examine two-hybrid interaction between the two proteins in both directions. The experimental and control interactions are listed in Supplementary Table 1. Colonies were initially patched onto an SC-Leu-Trp master plate before replica-plating onto phenotyping plates: (1) SC-Leu-Trp-Ura to test *URA3* activation; (2) SC-Leu-Trp-His + 3-amino-1,2,4-triazole (3-AT) to test *HIS3* activation; and (3) YPAD containing a filter for X-gal assay to test *lacZ* reporter activation.

An initial experiment to determine the appropriate concentration of the *HIS3*-inhibitor 3-AT used for the characterization of the activation of *HIS3* was performed by testing bait self-activation on SC-Leu-Trp-His supplemented with 0–100 mM 3-AT.

### Gene Expression Analysis

Gene expression analyses were conducted for *Ca4_TIFY4B* and selected putative downstream-regulated genes: *Ca11004* (GRF-Interacting Factor1; *CaGIF1*), *Ca27602* (GRF-Interacting Factor1-like; *CaGIF1L*), *Ca04724* (Growth Regulating Factor4; *CaGRF4*), and *Ca16785* (Growth Regulating Factor5-like; *CaGRF5L*). Whole roots and shoots at 9 days after sowing (days after sowing (DAS), *n* = 4) and youngest fully emerged leaves at 7 weeks (*n* = 3) were harvested for a Rupali/Genesis836 RIL50-derived pair of NILs (50.7) and snap-frozen in liquid nitrogen. The immature seed was sampled from an additional NIL pair derived from HIF 50.4.172 (*n* = 4). Frozen tissues were ground to a powder, and RNA was extracted from each sample using a Spectrum™ Plant Total RNA Kit (Sigma–Aldrich) with On-Column DNase I digestion. RNA quality and quantity were assessed using a NanoDrop spectrophotometer (NanoDrop Technologies Inc., Santa Clara, CA, United States). A 1 μg of each extracted RNA sample was used for cDNA synthesis using SuperScript III Reverse Transcriptase (Invitrogen, Carlsbad, CA, United States).

Quantitative real-time PCR (qRT-PCR) was performed in a Bio-Rad CFX Real-Time PCR System following the SsoAdvanced™ Universal SYBR^®^ Green Supermix protocol.^3^ Three technical replicates of each biological replicate were included. The transcript levels of each gene were normalized against three internal control genes, namely, *Ca31016* (Elongation factor 1-alpha; *EF1-a*), *Ca19204* (Glyceraldehyde 3-phosphate dehydrogenase, cytosolic-like; *GAPDH*), and *Ca31038* (Glucose-6-phosphate 1-dehydrogenase; *G6PD*) (Reddy et al., 2016; Amalraj, 2019; for primers, refer to Supplementary File 1). An absolute quantification method was employed to calculate the copy number using a standard curve derived from a set of standards containing 10^2^–10^7^ copies of the template. Raw data were analyzed using CFX Maestro Software version 2.2 (a suite of tools from CFX Real-Time PCR Systems).

### Identification and Structural Analysis of the Chickpea *TIFY* Gene Family

Fourteen sequences annotated as *TIFY* genes in chickpea (*CDC Frontier Ref V2.6.3*) and 31 other sequences (including isoforms) of the chickpea *TIFY* family (*CDC Frontier Ref V1.0*; Varshney et al., 2013) extracted from protein family PF06200 of the Pfam database (Mistry et al., 2021) were blasted against the *CDC Frontier Ref V2.6.3* assembly using the TBLASTN algorithm (*E*-value < 0.001) and protein-specific parameter BLOcks SUbstitution Matrix (BLOSUM62, Tong, 2013). All obtained protein sequences were subsequently searched for non-redundant hits and were individually scanned for the presence of constitutional motifs of the *TIFY* gene family using Pfam (Mistry et al., 2021) and HMMER (Potter et al., 2018). The identified chickpea *TIFY* genes were named according to their homology with *Arabidopsis*.

Phylogenetic trees were created *via* the Constraint-based Multiple Alignment Tool (COBALT; Papadopoulos and Agarwala, 2007) by adapting neighbor-joining (Saitou and Nei, 1987) and using full-length amino acid sequences (Supplementary File 2) of Ca4_TIFY4B and its homologs among various species. Multiple Expectation maximization for Motif Elicitation (MEME; Bailey et al., 2009) was used to create a block diagram of motifs for the orthologs of Ca4_TIFY4B. A search on the MEME suite was executed to identify distinctive motifs with the following parameters: (1) width of optimum motif ≥6 and ≤50; (2) maximum number of most significant motifs to identify = 3.

## Results

### Fine Mapping of *Ca4_Vqtl*

To identify robust component traits for fine mapping, a phenotypic profiling experiment for a range of vigor-related traits was performed using six *Ca4_Vqtl* Rupali/Genesis836 RIL-derived NIL pairs grown under shade house conditions (Supplementary File 3). Among the studied traits, leaf size, seed number, seed size, and 100SDW showed complete statistically significant segregation across all NIL pairs. Other traits, including subjective and objective (FGCC) measures of whole plant vigor, did not show clear differences between genotypes (Supplementary File 3). Leaf size, seed number, seed size, and 100SDW traits were selected for the fine-mapping analysis of 15 HIFs under glasshouse conditions: 11 HIFs segregated significantly for each trait (Supplementary Table 2). Leaf size, seed size, and 100SDW were significantly positively correlated with each other (*r* = 0.66 to 0.87, *P* < 0.01) but were negatively correlated with seed number (*r* = −0.42 to −0.75, *P* < 0.01).

**FIGURE 1:**
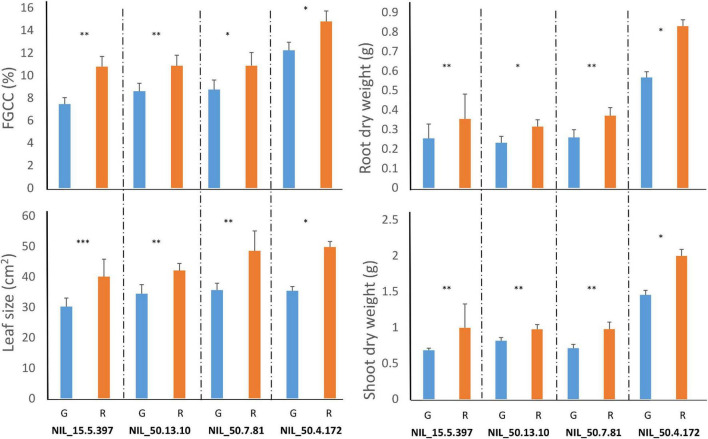
Phenotypic analysis in four heterogeneous inbred family (HIF)-derived near-isogenic line (NIL) pairs harvested after 4 weeks of growth in a greenhouse. G, Genesis836 allele and R, Rupali allele at *Ca4_Vqtl*. FGCC, Fractional Green Canopy Cover (%) using imaging analysis with the Canopeo application (Oklahoma State University, Stillwater, OK, United States; https://canopeoapp.com/). Student’s *t*-test was used to identify significant differences between lines from each NIL pair (*n* = 4). Student’s *t*-test significance codes: *P < 0.05, ^**^P < 0.01, and ^***^P < 0.001.

**FIGURE 2:**
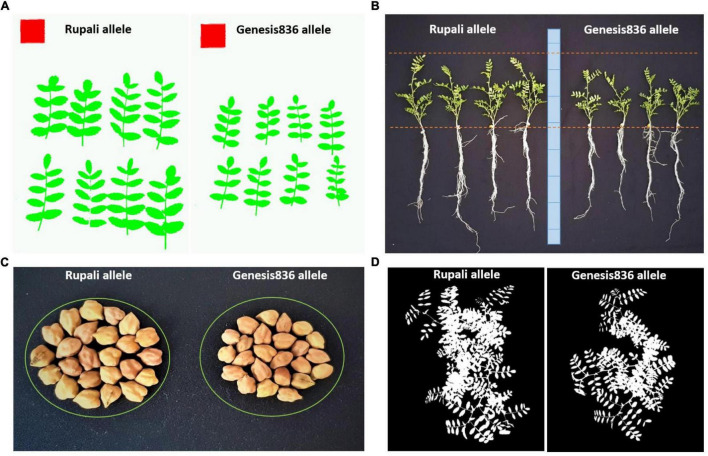
Representative images showing differences in panel **(A)** leaf size, **(B)** biomass, **(C)** seed size, and **(D)** canopy size for plants carrying the Rupali or Genesis836 *Ca4_Vqtl* allele (NIL_50.7.81). Leaf images were taken using the Easy Leaf Area app (Easlon and Bloom, 2014), and canopy images were taken using the Canopeo application (Oklahoma State University, Stillwater, OK, United States; https://canopeoapp.com/).

To further investigate component traits associated with *Ca4_Vqtl*, four HIF-derived NIL pairs were grown under greenhouse conditions and harvested after 4 weeks of growth. Significant differences for FGCC, leaf size, and root and above-ground biomass at harvest were observed in all NIL pairs contrasting for *Ca4_Vqtl* (Figure 1). The observation that FGCC was significantly different between contrasting NIL pairs in this experiment but not in shade house conditions suggests that the expression of some vigor-related traits is influenced by the growing environment. Plants carrying the Rupali *Ca4_Vqtl* allele had more canopy cover (22.15% increase in FGCC), larger leaves (24.57% increase), and greater root and shoot dry weights (increases of 29.12 and 25.65%, respectively) compared to plants carrying the Genesis836 allele (Supplementary File 4). Representative images of plant organ size differences observed across the NIL phenotyping experiments are illustrated in Figure 2.

**FIGURE 3:**
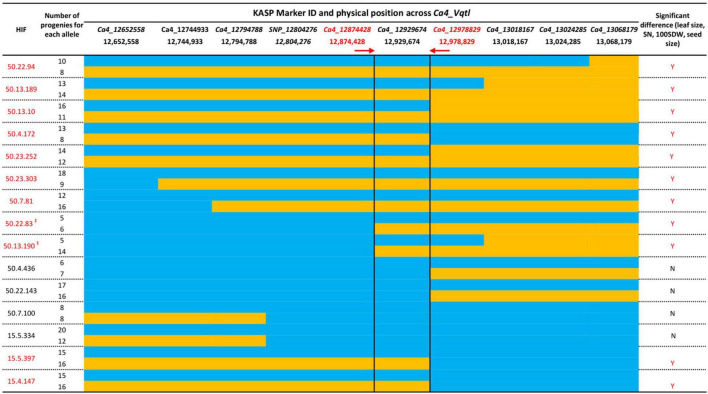
Fine-mapping analysis of *Ca4_Vqtl* in Rupali/Genesis836 recombinant families. Fifteen HIFs with recombination within *Ca4_Vqtl* were phenotyped in greenhouse conditions in 2020 and 2021. Ten Kompetitive Allele-Specific PCR (KASP)-single-nucleotide polymorphism (SNP) markers were used to genotype and identify recombinant breakpoints. Orange color indicates Rupali (R) allele, while blue color indicates Genesis836 (G) allele. Red text highlights HIFs where significant differences were observed between allelic classes for leaf size, seed number (SN), average weight of 100 seed weight (100SDW), and seed size by Student’s *t*-test (*P* < 0.05). Red arrows show the reduced interval between markers (in red) *Ca4_12874428* and *Ca4_12978829* (104.4 kb). (‡) Indicate lines phenotyped in 2021 under similar growing condition to 2020. The phenotyping data used for fine mapping is presented in Supplementary Table 2.

Recombination events across the *Ca4_Vqtl* (12.65–13.06 Mb) were identified using 10 KASP markers specific to SNPs identified between Rupali and Genesis836 across the region (Figure 3). Association analysis using HIF phenotypic and genotypic data demonstrated that the genomic interval between KASP markers *Ca4_12874428* and *Ca4_12978829* was consistently linked to phenotype (Figure 3). This interval spans 104.4 kb and contains 10 annotated genes according to *CDC Frontier Ref V2.6.3* (Supplementary Table 3). These 10 genes were examined for putative association with vigor and component traits. To investigate if the difference in vigor contributed by the region was related to transcript dosage levels, the differential expression between the second youngest fully expanded leaves of 20-day-old plants of Genesis836 and Rupali was examined using previously acquired data (Khan, 2016; NCBI Accession PRJNA798198; Supplementary Figure 1). No significant difference in expression for the 10 genes located within the critical 104.4 kb *Ca4_Vqtl* region was observed.

To determine whether the vigor effect at the *Ca4_Vqtl* locus is the result of sequence difference, we interrogated previously acquired sequence capture data for the RIL parents Rupali and Genesis836, which spanned genomic sequence at an average read depth of 100× across the broader *Ca4_Vqtl* region (Nguyen et al., 2021). SNPs associated with four genes were identified: *SNP_12881281* in the intron of *Ca11857* (Aldo/keto reductase family oxidoreductase); *SNP_12903671* in the promoter of *Ca11871* (Heat shock transcription factor A3); *SNP_12922166* in the intron of *Ca11865* (1,2-dihydroxy-3-keto-5-methylthiopentene dioxygenase); and *SNP_12929674* in the exon of *Ca11869* (*TIFY4B*-like isoform X1; *Ca4_TIFY4B*). *SNP_12929674* was the only exonic SNP identified in coding sequences.

The exonic *SNP_12929674* was previously used to validate the presence of *Ca4_Vqtl* in three different chickpea diversity panels (Nguyen et al., 2021), where it was significantly associated with both vigor and seed size across the panels (*P* < 0.001). Further investigation of *SNP_12929674* in *Ca4_TIFY4B* showed that it is non-synonymous and results in a non-conservative amino acid substitution between isoleucine (I) and serine (S) (Pechmann and Frydman, 2014). *Ca4_TIFY4B* belongs to a family of transcription factors known as TIFY, and it is orthologous to PEAPOD (*PPD1*/*PPD2*) in *Arabidopsis thaliana* (White, 2006, 2017; Liu et al., 2020) and Big Seed 1 (*BS1*) in *Medicago truncatula* (Ge et al., 2016), which control organ size and shape. Based on the accumulated evidence, *Ca4_TIFY4B* was a strong candidate at the delineated *Ca4_Vqtl.*

### *TIFY* Gene Family in Chickpea

To identify any other paralogs of *Ca4_TIFY4B* and further explore the *TIFY* gene family in chickpea, protein BLASTs of the chickpea genome assembly (*CDC Frontier Ref V2.6.3*) and HMM searches identified eighteen *CaTIFY* genes (Table 1). The *TIFY* genes were named according to their similarity with *Arabidopsis* sequences, and their protein sequences are provided in Supplementary File 2. The *TIFY* gene family contains the core motif TIF[F/Y]XG and can be classified into four subfamilies, namely, TIFY, JAZ, ZML, and PPD, depending on whether they contain additional domains/motifs (Vanholme et al., 2007; Bai et al., 2011). Proteins with only the TIFY (PF06200) domain are classified as the TIFY subfamily (Vanholme et al., 2007); proteins with both the TIFY and jasmonate ZIM domains (Jas, PF09425) are classified as the JAZ subfamily (Staswick, 2008); proteins containing TIFY, PPD domains, and a truncated Jas domain are classified as the PPD subfamily (White, 2006); and proteins containing the TIFY domain and the CCT (PF06203) and/or ZML*/*GATA (PF00320) domain are classified as the ZML subfamily. Among the 18 *CaTIFY* genes, 10 belong to the JAZ subfamily (Table 1). Of these, nine JAZ were previously reported by Singh et al. (2015). Two *CaTIFY* genes, namely, *Ca20678* and *Ca07271*, belong to the TIFY subfamily. Five other *CaTIFY* genes, namely, *Ca05650*, *Ca21020*, *Ca21022*, *Ca02171*, and *Ca29422*, are grouped into the ZML subfamily. *Ca4_TIFY4B* (*Ca11869*; Table 1), which carries PPD, TIFY, and Jas domains, is the only *CaTIFY* gene encoding for a PPD protein in chickpea.

**TABLE 1:** Structural and coding details of chickpea *CaTIFY* genes.

TIFY subfamily	Gene name	Ca	Gene ID (V2.6.3)	Physical position (bp)	No. of exon	CDS length (bp)	aa length	Domains
TIFY	*DEG5*	Ca1	Ca07271	3,985,021–4,021,646	15	1,908	636	TIFY
	*TIFY8*	Ca5	Ca20678	16,910,106–16,914,119	6	1,266	422	TIFY
JAZ	*TIFY10A-like*	Ca1	Ca08916	21,469,342–21,471,946	4	888	296	TIFY, Jas
	*TIFY3B-like*	Ca1	Ca09015	22,832,165–22,834,911	5	636	212	TIFY, Jas
	*TIFY6A*	Ca4	Ca13423	35,172,993–35,177,878	8	1,203	401	TIFY, Jas
	*TIFY5A-like*	Ca6	Ca01899	729,424–726,914	3	411	137	TIFY, Jas
	*TIFY6B-like*	Ca6	Ca03206	13,091,228–13,094,228	7	1,056	352	TIFY, Jas
	*TIFY11B*	Ca7	Ca16812	23,640,068–23,642,598	5	723	241	TIFY, Jas
	*TIFY10A-like*	Ca7	Ca18389	41,170,284–47,171,756	5	675	225	TIFY, Jas
	*TIFY3B*	Ca7	Ca18427	47,516,849–47,519,317	6	606	202	TIFY, Jas
	*TIFY10A-like*	Ca8	Ca00368	2,613,103–2,614,783	4	525	175	TIFY, Jas
	*TIFY6B*	Ca8	Ca01249	10,722,096–10,729,380	9	1,278	426	TIFY, Jas
**PPD**	***TIFY4B-like***	**Ca4**	**Ca11869**	**12,931,952–12,926,557**	**9**	**1,002**	**334**	**PPD, TIFY, Jas**
ZML	*TIFY1*	Ca2	Ca29422	10224865–10229324	7	918	306	TIFY, CCT, ZML
	*TIFY2B*	Ca5	Ca21020	19,896,795–19,902,475	10	1,062	354	TIFY, CCT, ZML
	*TIFY1*	Ca5	Ca21022	19,889,422–19,893,881	8	912	304	TIFY, CCT, ZML
	*TIFY2A*	Ca6	Ca05650	49,145,432–49,149,866	7	900	300	TIFY, CCT, ZML
	*TIFY2A*	Ca6	Ca02171	3,147,186–3,151,427	11	1,023	341	TIFY, CCT, ZML

*Ca, chickpea chromosome; CDS, coding sequence; aa, amino acid.*

*Proteins with only the TIFY (PF06200) domain are classified as the TIFY subfamily; proteins with both the TIFY and jasmonate ZIM domains (Jas, PF09425) are classified as the JAZ subfamily; proteins (in bold) containing TIFY, PPD domains, and a truncated Jas domain are classified as PPD subfamily; and proteins containing the TIFY domain and the CCT (PF06203) and/or ZML/GATA (PF00320) domain are classified as the ZML subfamily.*

### *Ca4_TIFY4B* Encodes a PEAPOD Protein and Interacts With *CaNINJA*

**FIGURE 4:**
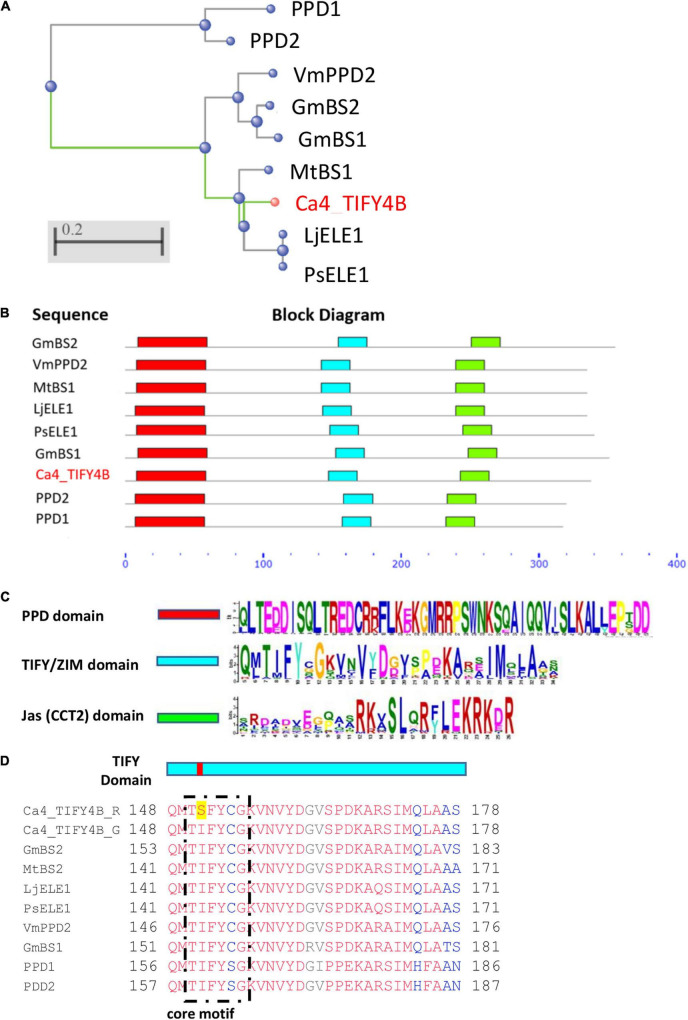
**(A)** Phylogenetic relationship of Ca4_TIFY4B protein sequence with orthologs from other plant species: *G. max*, BS1, BS2, *V. mungo*, VmPPD; *M. truncatula*, BS1; *P. sativum*, ELE1; *Lotus japonicus*, ELE1; *A. thaliana*, PPD1 and PPD2. Data were analyzed *via* Constraint-based Multiple Alignment Tool [COBALT, Papadopoulos and Agarwala (2007)]. The tree was created using a neighbor-joining method (Saitou and Nei, 1987). Cluster distance scale is shown on the gray bar (0.2) **(B)** Putative conserved domain structure in the CaTIFY4B protein and its homologs in other species, assessed using the Multiple Expectation maximization for Motif Elicitation (MEME) web server (https://meme-suite.org/meme/). The blue scale indicates amino acid sequence length; the color blocks represent the positions of domains. **(C)** The consensus sequences of conserved domains for TIFY, Jas (CCT2), and PPD from TIFY family proteins were created using MEME. **(D)** Sequence alignment across the TIFY domain. The isoleucine/serine (I/S) substitution in Ca4_TIFY4B_R is highlighted.

*Ca4_TIFY4B* has nine exons and encodes a TIFY family transcription factor (334 amino acids), with high overall sequence similarity (Figure 4A) and major PPD, TIFY, and Jas domains (Figures 4B–C) characteristic of dicot *PPD* genes (TIFY sub-family). The amino acid substitution (I/S) found in the Rupali Ca4_TIFY4B protein is located inside the core TIFYCG motif of the TIFY domain (Figure 4D), whereas Genesis836 carries a version similar to the TIFY domain of other characterized PPD proteins. Previously, Ca4_TIFY4B orthologs were reported to interact with other protein partners including NINJA to form a complex regulating expression of transcription factors and/or downstream genes in *Arabidopsis* and *Medicago* (Ge et al., 2016; Baekelandt et al., 2018). It was shown that the core motif “TIFYAG” is the key region of the TIFY domain that interacts with AtNINJA in *Arabidopsis* (Pauwels et al., 2010).

**FIGURE 5:**
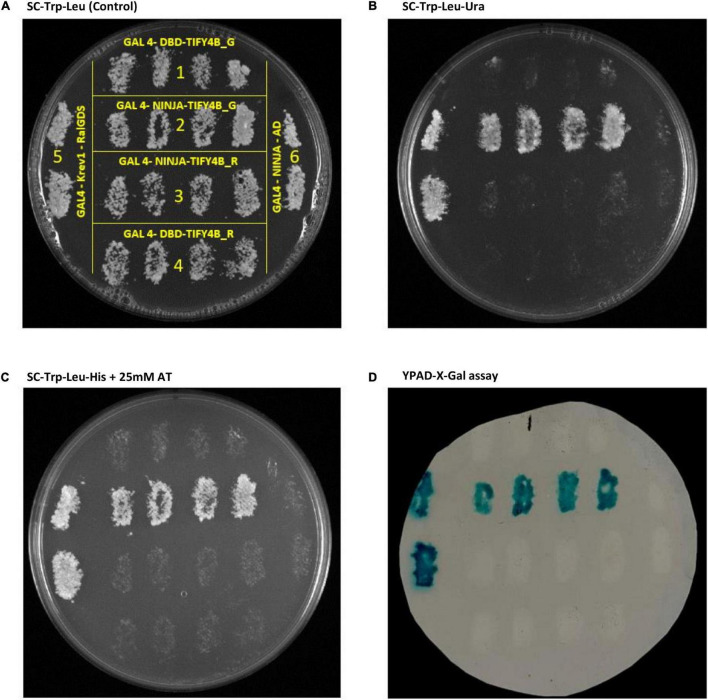
*Saccharomyces cerevisiae* Y2H analysis of protein-protein interactions between CaNINJA and Rupali or Genesis836 alleles of CaTIFY4B. **(A)** Control plate. **(B)** Test for *URA3* reporter gene activation. **(C)** Test for *HIS3* reporter gene activation. **(D)** Test for *lacZ* reporter gene activation. Plates in panels **(B–D)** were replica plated from plate **(A)**; growth on these plates indicates a positive protein-protein interaction. Each patch is derived from a single transformed colony; patches for 1, 4, and 6 are negative controls while 5 (GAL4-Krev1-RalGD) is a strong positive control. Details for each of the construct interactions are provided in Supplementary Table 1.

To test the impact on protein dimerization of the Rupali SNP variant inside the TIFY motif, Y2H interaction experiments for Ca4_TIFY4B and CaNINJA (*Ca01446*) were implemented in two ways using entire coding sequences: (1) CaNINJA as bait and TIFY4B_R/TIFY4B_G as preys (Figure 5) and (2) TIFY4B_R/TIFY4B_G as baits and CaNINJA as prey (Supplementary Figure 2). The CaNINJA sequences from Rupali and Genesis836 were identical (Supplementary File 5). Strong interaction between NINJA and TIFY4B_G was observed across the phenotyping plates when NINJA was used as bait (Figure 5), while weaker interaction between the two was observed only on the *HIS3* reporter gene activation plate, when TIFY4B_G was used as bait (Supplementary Figure 2). No interaction was found between NINJA and TIFY4B_R when these tests were performed in either direction (Figure 5 and Supplementary Figure 2).

### Expression of Potential Downstream Target Genes of Ca4_TIFY4B

**FIGURE 6:**
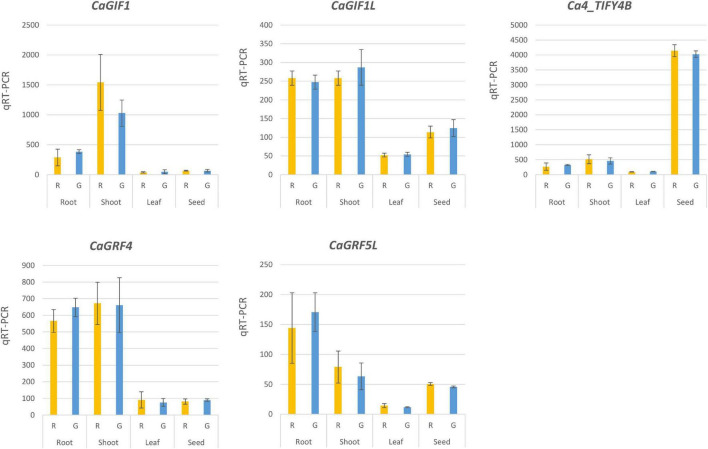
Absolute quantitative real-time PCR (qRT-PCR) analysis of expression of chickpea *GIF1*, *GIF1L*, *GRF4*, *GRF5L*, and *Ca4_TIFY4B* in NILs carrying contrasting *Ca4_TIFY4B* alleles, Rupali (R) or Genesis836 (G). NILs were derived from RIL50, heterozygous across the vigor region *Ca4_Vqtl*. Gene expression was analyzed for whole shoot and root harvested at 9 days after sowing, youngest fully emerged leaves sampled at 7 weeks after sowing, and developing seed. Means ± SD are shown for *n* = 3 or 4. There were no significant differences in expression for any of the tested genes in these tissues (*P* > 0.05).

The residue change identified in the TIFY domain of the Rupali Ca4_TIFY4B variant abolished interaction with CaNINJA in a Y2H assay. To determine the impact on the expression of potential target genes of the transcriptional complex, candidates were chosen based on proposed target genes for the Ca4_TIFY4B ortholog *MtBS1* in *Medicago* (Ge et al., 2016) and *PPD1*/*PPD2* in *Arabidopsis* (Horiguchi et al., 2005; Lee et al., 2009; Kawade et al., 2013). We examined expression in four tissue types, namely, young shoot and root (harvested at nine DAS), youngest fully emerged leaf, and developing seed, for *Ca4_TIFY4B*, and four downstream target genes of the Ca4_TIFY4B-CaNINJA complex, namely, *CaGIF1*, *CaGIF1L*, *CaGRF4*, and *CaGRF5L*. All genes showed differential tissue-specific expression patterns. *Ca4_TIFY4B* was most highly expressed in developing seed. In contrast, the four candidate downstream genes were more highly expressed in young shoot and root than in youngest fully emerged leaves or developing seed (Figure 6). Neither *Ca4_TIFY4B* nor the selected downstream target genes were significantly differentially expressed in the contrasting NILs chosen for analysis (Figure 6). This outcome contradicted the hypothesis that the Ca4_TIFY4B_R variant contributes to increased vigor through the inability to interact with the CaNINJA partner to upregulate the expression of the genes known to regulate organ growth (Ge et al., 2016).

## Discussion

Vigor has been identified as a high-priority target in pulse crop breeding programs. An understanding of the genetics of vigor and the development of robust genetic markers for the trait would allow breeders to select early stage breeding materials. In this study, we fine-mapped a vigor QTL, *Ca4_Vqtl*, which colocates with a region for seed size in a population of Rupali/Genesis836 chickpea RILs (Nguyen et al., 2021). The QTL spans 12.65–13.06 Mb (*CDC Frontier Ref V2.6.3*) and overlaps a “hotspot” QTL region identified independently for a biparental population that was linked to drought tolerance, vigor, and seed size traits (Varshney et al., 2014; Jaganathan et al., 2015; Kale et al., 2015; Singh et al., 2016; Sivasakthi et al., 2018). The analysis of key recombinant families in this study has allowed us to narrow the interval to 104.4 kb and revealed the regulatory gene *Ca4_TIFY4B* as a strong candidate at *Ca4_Vqtl* for vigor through the regulation of organ size. In our study, the measurement of leaf and seed size together with 100SDW and seed number were effectively used as robust measurements for trait/marker associations as they segregate with genotype at *Ca4_Vqtl* in both glasshouse and shade house conditions (Supplementary File 3 and Supplementary Table 2). In glasshouse conditions, we also observed segregation for root dry weight at 4 weeks in the NIL pairs (Figure 1). This supports previous findings that the “*QTL hotspot*” controls root-based traits (Varshney et al., 2014; Kale et al., 2015). Our study supports the hypothesis of a pleiotropic effect of the *Ca4_Vqtl* region in controlling organ size, including that of leaves, roots, and seeds.

Of the four SNPs reinvestigated within the refined *Ca4_Vqtl* interval, only one was found within a coding region and likely to have a major impact on gene function. Of the 10 genes located inside *Ca4_Vqtl*, *Ca4_TIFY4B* contains a non-synonymous SNP inside the fourth exon which leads to a non-conservative amino acid substitution (I/S) in the core TIFYCG motif sequence of its TIFY domain (Vanholme et al., 2007). *Ca4_TIFY4B* is the single TIFY ortholog of the PPD subfamily found in chickpea and is orthologous to *PPD1*/*PPD2* in *A. thaliana* and *BS1* in *M. truncatula*, which both control organ size and shape (White, 2006; Gonzalez et al., 2015; Ge et al., 2016). *ppd1-2 ppd2-*cr and *ppd1-cr ppd2-1* double mutants generated in *Arabidopsis* had greater individual SWs (Liu et al., 2020), whereas the overexpression of *PPD1* or *PPD2* (Δ*ppd-*deletion complementation) resulted in a reduction of SW (White, 2017). Ge et al. (2016) reported that deletion and downregulation of *PPD* orthologs in *Medicago* (*MtBS1*) and soybean (*GmBS1*), respectively, led to significant increases in leaf and seed size. In black gram [*Vigna mungo* (L.) Hepper], a loss-of-function mutation (8-bp deletion in the sixth exon) in *VmPPD* (*mog* mutant), led to dramatic increases in leaf size, biomass, and seed size (Naito et al., 2017). In pea (*P. sativum*), *PsELE1* mutants displayed contrasting phenotypes to the wild type, with enlarged seeds, leaves, symmetrical lateral, and ventral petals (Li et al., 2019). These functional studies of *Ca4_TIFY4B* orthologs reveal a conserved function and suggest a similar role for *Ca4_TIFY4B* in controlling tissue organ size, i.e., component traits of vigor in chickpea.

Previously, TIFY domains of PPD proteins were reported to mediate homo- and heterodimerization between different TIFY proteins and with other protein partners, including NINJA (Chini et al., 2009; Chung and Howea, 2009; Pauwels et al., 2010; Gonzalez et al., 2015). In *Arabidopsis*, it was shown using Y2H and an AtJAZ1 deletion series that AtNINJA was only capable of binding JAZ1 fragments containing a TIFY motif, specifically the “TIFYAG” sequence (Pauwels et al., 2010). The SNP we identified in the TIFY domain of Ca_TIFY4B results in a change from the conserved TIFY domain of TIFYCG to TSFYCG (Figure 4D). As the TIFY domain is known to mediate interactions with NINJA proteins, we tested the interaction of the two versions, Ca4_TIFY4B from Genesis836 (Ca4_TIFY4B_G; smaller leaf and seed) and Rupali (Ca4_TIFY4B_R; bigger leaf and seed), with CaNINJA. We found that CaNINJA interacted strongly with the Ca4_TIFY4B_G version, enabling yeast growth, whereas Ca4_TIFY4B_R assays resulted in no observable growth under our conditions (Figure 5). This is likely attributed to the non-conservative amino acid substitution (I/S) in the core TIFYCG motif (Figure 4).

The complexes formed by orthologs of Ca4_TIFY4B, CaNINJA, and other PPD partners are proposed to suppress the expression of organ size regulatory genes in plants (Schneider et al., 2021). In *Medicago MtBS1* loss-of-function mutants, several proposed target genes involved in primary cell proliferation (*MtGIF1*, *MtGIF2*, *MtGRF1*, and *MtGRF5*) were found to be upregulated, suggesting that the native functional complex suppresses their expression (Ge et al., 2016). In this study, we initially hypothesized that the inability of Ca4_TIFY4B_R to interact with CaNINJA, which is caused by the variant SNP in the TIFY domain, would disrupt the function of the whole PPD complex and result in the increased expression of *CaGIF1*, *CaGIF1L*, *CaGRF4*, and *CaGRF5L*. However, we observed no differences in transcript expression in young shoots and roots, youngest fully emerged leaves, or developing seed of selected pairs of NILs (Figure 6). Even though the findings from our expression study contradict the *Medicago* study, they are similar to findings in *Arabidopsis* where *PPD1/PPD2* knockdown lines showed a size-increase phenotype yet the expression of *AtGIF1* and *AtGRF5* were not altered (Gonzalez et al., 2015). Even where *PPD1*/*PPD2* were dramatically downregulated to 13 and 40% of the wild-type gene expression levels, respectively (Gonzalez et al., 2010), the regulating function of the PPD complex was partly retained, and hence, the expression of downstream genes was not observably affected in *Arabidopsis* (Gonzalez et al., 2015). Based on these results, we propose that this could also be the case with the natural mutation found in *Ca4_TIFY4B*. In the native chickpea background, the SNP in *Ca4_TIFY4B* might result in a decrease in the binding affinity between Ca4_TIFY4B and CaNINJA, rather than a complete loss of function as observed in the Y2H experiment. *In planta*, the functional impairment of the Ca4_TIFY4B_R/NINJA/protein partner complex rather than a complete abrogation of its regulatory function would not be expected to impact the regulation of these potential downstream genes as strongly as observed in dramatically truncated *PPD* mutants generated in other species (Ge et al., 2016; Liu et al., 2020). This is also reflected in the less pronounced increase in organ size of our plants carrying the natural *Ca4_TIFY4B_R* variant compared with other reports of plants carrying *PPD* knockout complete loss-of-function alleles. We only observed increases in leaf and seed size by 39 and 24%, respectively, in *Ca4_TIFY4B_R* genotypes, compared to the increases of 100 and 70% in black gram *mog* lines (Naito et al., 2017), 225 and 49% in the *Medicago mtbs1-1* lines (Ge et al., 2016), and 53% (for seed size) in *Arabidopsis* Δ*PPD* (Liu et al., 2020).

The significant increases in the shoot, biomass, leaf, fruit, and seed size in eudicots, including legume plants, upon natural mutation or genetic engineering of *PPD* proteins and their partners imply great potential for breeding from a biotechnological point of view (Gonzalez et al., 2015; Ge et al., 2016; Naito et al., 2017; Kanazashi et al., 2018; Li et al., 2019; Liu et al., 2020; Swinnen et al., 2020). However, targeting genes that act in multiple plant organs or at distinct developmental stages, as demonstrated for the *PPD*, may have undesirable pleiotropic effects. In this study, we showed that the natural SNP variant in *Ca4_TIFY4B* could lead to increases in leaf size, seed size, and 100SDW by 39, 24, and 35%, respectively, but also resulted in a decrease of seed number per plant by approximately 39% (Supplementary File 4). A similar trade-off between these traits was reported in black gram and soybean (Naito et al., 2017; Kanazashi et al., 2018). Nevertheless, the screening of *GmBS1* and *GmBS2* knockdown transgenic lines in soybean found the presence of transgenic plants with increased seed size and maintenance of seed number; this suggests a potential for regulating *GmBS1* and *GmBS2* expression levels to reduce the seed size/number trade-off and to enhance total SW (Naito et al., 2017). While our findings from pot-grown plants of *Ca4_Vqtl* NILs suggested that a reduction in total SW is associated with the increase in vigor and seed size (Supplementary File 3), Bharadwaj et al. (2021) reported that introgression of the *QTL hotspot*/*Ca4_Vqtl* (including *Ca4_TIFY4B_R* from the high vigor/big seed parent ICC4958) into three chickpea elite cultivars enhanced yield by 16% under rainfed conditions in India. The *Ca4_TIFY4B_R* variant (*SNP_12929674*) was recently found to be significantly strongly associated with both vigor and seed size in three chickpea diversity panels, including Australian varieties and breeding lines (Nguyen et al., 2021). However, the *Ca4_TIFY4B_G* variant is almost three times more prevalent than the *Ca4_TIFY4B_R* variant in Australian commercial chickpea varieties (Supplementary File 6). This suggests that the *Ca4_TIFY4B_G* variant might have been passively selected to mitigate the potential impact of a seed size/number trade-off for the improvements of yield under Australian growing conditions. Further studies using *Ca4_Vqtl* NIL pairs in various Australian environments are needed to confirm this. However, while the manipulation of this *PPD* gene might not necessarily achieve consistently higher yields, the larger seed size could attract a price premium, offset a yield disadvantage (relative to other crops), and improve the commercial value of leguminous crops in the farming system.

This study has revealed the gene *Ca4_TIFY4B* as the strongest candidate inside the narrowed *Ca4_Vqtl* interval that controls vigor-related traits in chickpea. We have identified a novel, natural variant of a plant *PPD* gene that results in moderate increases in seed size, leaf size, and other plant organs. We described a robust gene-derived molecular marker to be used for selection for seed and organ size in chickpea. This will assist chickpea breeders in developing effective strategies to significantly increase plant vigor and also manipulate seed size and yield.

## Data Availability Statement

The original contributions presented in the study are included in the article/Supplementary Material, further inquiries can be directed to the corresponding author.

## Author Contributions

DN, JEH, JH, and TS designed and conceived the study. JEH and DN conducted phenotyping and Y2H assays. JH and DN conducted the qPCR assays. DN collected data and drafted the manuscript. All authors provided feedback on the manuscript and read and approved the final manuscript.

## Conflict of Interest

The authors declare that the research was conducted in the absence of any commercial or financial relationships that could be construed as a potential conflict of interest.

## Publisher’s Note

All claims expressed in this article are solely those of the authors and do not necessarily represent those of their affiliated organizations, or those of the publisher, the editors and the reviewers. Any product that may be evaluated in this article, or claim that may be made by its manufacturer, is not guaranteed or endorsed by the publisher.

## Footnotes

1
http://www.biogro.com.au/

2
https://canopeoapp.com/

3
https://www.bio-rad.com/

## Supplementary Material

Supplementary File 1 Primer sequences.

Supplementary File 2 FASTA of protein sequences from this study.

Supplementary File 3 Recombinant inbred line (RIL)-derived near-isogenic lines (NILs) phenotyping.

Supplementary File 4 Heterogeneous inbred families (HIFs) and HIF-derived near-isogenic lines (NILs) phenotyping variation.

Supplementary File 5 Rupali and Genesis836 *Ca4_TIFY4B* and *CaNINJA* sequences.

Supplementary File 6 Distribution of *Ca4_TIFY4B* allele in Australian varieties.
