# Supplementary material for: Fine Mapping of a Vigor QTL in Chickpea (Cicer arietinum L.) Reveals a Potential Role for Ca4_TIFY4B in Regulating Leaf and Seed Size
Source: Front Plant Sci. 2022 Feb 24;13:829566. doi: 10.3389/fpls.2022.829566 (PMC8908238; doi:10.3389/fpls.2022.829566)
Supplement: Supplementary File 1 — Primer sequences. [file Data_Sheet_1.zip › Supplementary Material/Supplementary Figures S1 - S2.pdf]

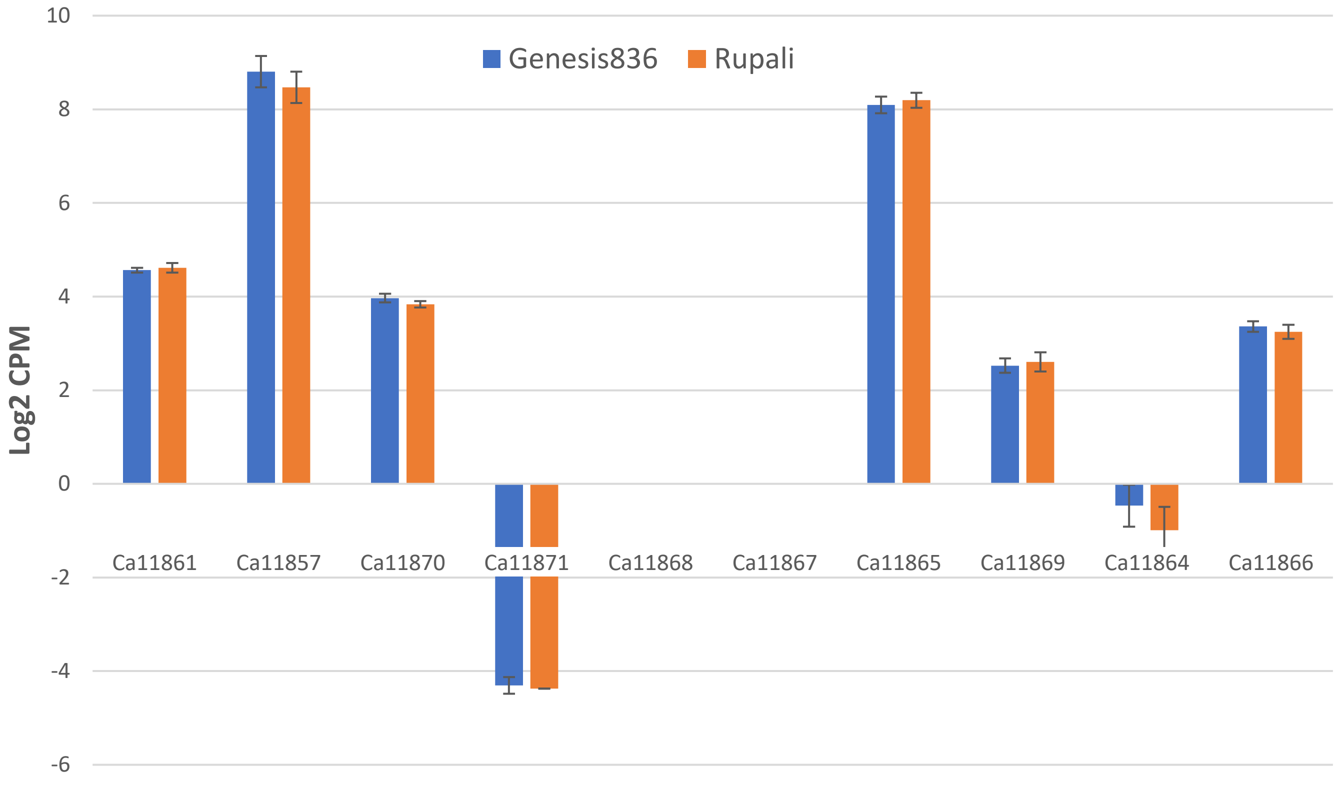

Supplementary Fig. S1. Expression analysis of genes in the narrowed *Ca4_Vqtl* region in Rupali and Genesis836. Data were extracted from Khan (2016) from RNAseq in second youngest fully-expanded leaf of 20-day-old plants. Log2CPM: Log_2 of read counts per million. Shown are means ± SD for n = 6. *Ca11868* and *Ca11867* were not expressed in this tissue. Ca11861, *Ca11857*, *Ca11870*, *Ca11871*, *Ca11868*, *Ca11867*, *Ca11865*, *Ca11869*, *Ca11864*, and *Ca11866* were not differentially expressed between Rupali and Genesis836 (P > 0.05).

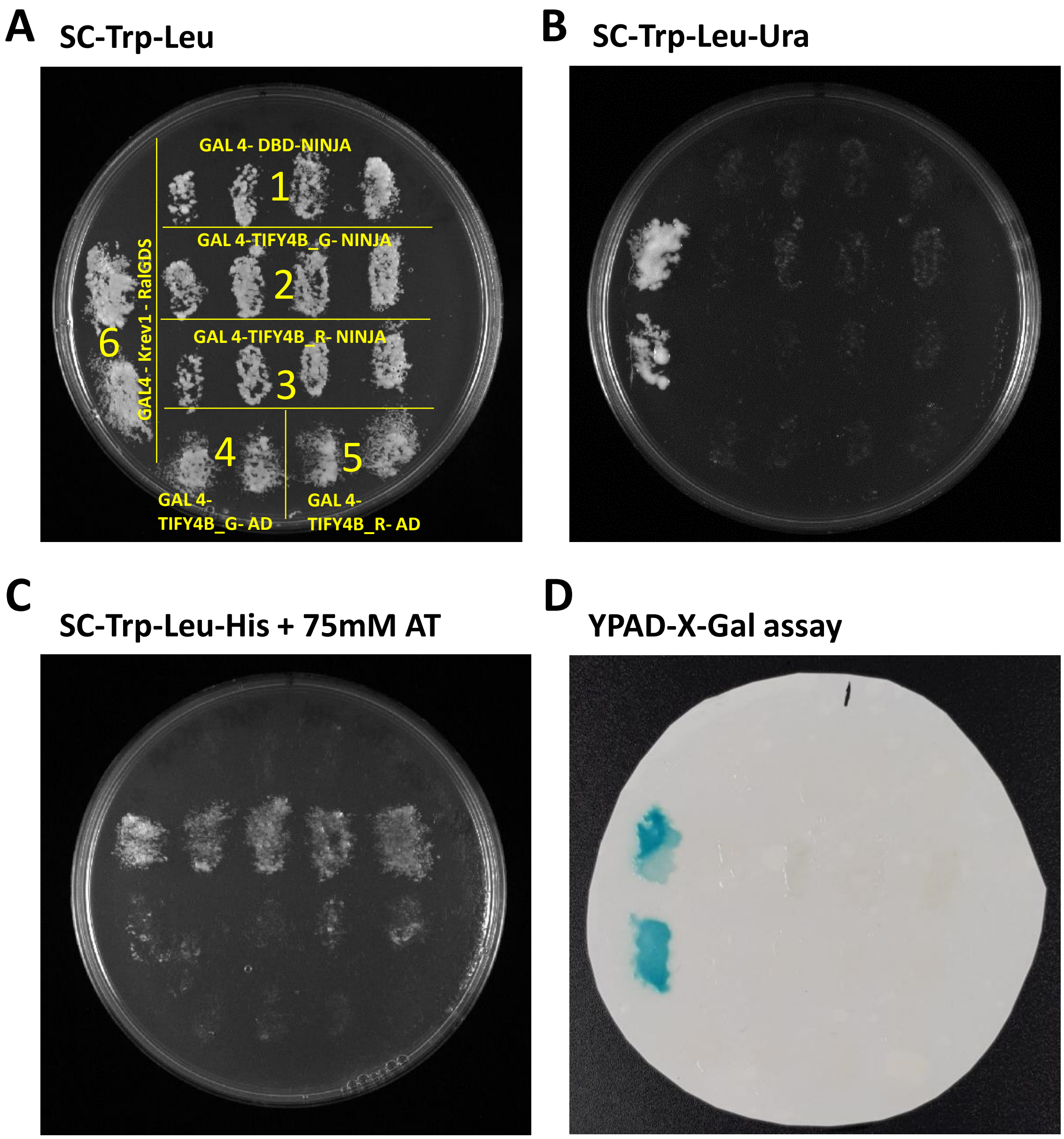

Supplementary Fig. S2. ***S. cerevisiae*** **Y2H analysis of protein-protein interactions between Rupali or Genesis836 alleles of CaTIFY4B and CaNINJA . (A)** Control plate. **(B)** Test for *URA3* reporter gene activation. **(C)** Test for *HIS3* reporter gene activation. **(D)** Test for *lacZ* reporter gene activation. Plates in B, C, and D were replica plated from plate A; growth on these plates indicates a positive protein-protein interaction. Each patch is derived from a single transformed colony.; patches for 1, 4 and 5 are negative controls while 6 (GAL4-Krev1-RalGD) is a strong positive control. Details for each of the construct interactions are provided in Supplementary Table 1.
