# Supplementary material for: Fine Mapping of a Vigor QTL in Chickpea (Cicer arietinum L.) Reveals a Potential Role for Ca4_TIFY4B in Regulating Leaf and Seed Size
Source: Front Plant Sci. 2022 Feb 24;13:829566. doi: 10.3389/fpls.2022.829566 (PMC8908238; doi:10.3389/fpls.2022.829566)
Supplement: Supplementary File 1 — Primer sequences. [file Data_Sheet_1.zip › Supplementary Material/Supplementary File S2. FASTA of protein sequences from this study..DOCX]

**Protein sequence of chickpea *TIFY* gene family**

>Ca1_Ca07271_TIFY_Subfamily

MELLYLLCSIVFTSFTSLILSLILPFHALLRRLGSSRATSSASNDGAEPITLYEGTVYHQRRHPVHHSFQ

YQVRYALIDLDRAHHAPHGHLSPDEARKITDTDGPILLLTIPSSVGYEQNPLSVYYCYEVEDSATRLKKC

IAEVTNTPWAERVTFIFNPHSDLVAKALHVSPFMDMLGSWNIKASDPGENLTLSISVHHPEFGNYFTASL

KAKKLRSSSSLDHAVFFWLMPHKIAVWIYWHALKLWWKNVQFIQHPRYMIPTYRDEALIRDRKLQCCVLS

DNNRHIQQRGSDRDCLAESSPRDRRVEEEDHSDKRLTTQNQQQPLTIFYDGKICVTDVTEFQAKSILMMA

NKKLQETILFTSTLSLASSFNFLNLTSKLPLSLSQLLQDDELQQQEDLVVNLFQEASPSVVFIKDIELTK

VPENSSNEVLLNEDIDAKVEGTGSGFIWDKFGHIVTNYHVVAKLATDTSGLQRCKVFLVDAKGNSFSREG

KLIGFDPAYDLAVLKVDVDGYEIKPVVLGDSKNLHVGQSCFAIGNPYGYENTLTTGVVSGLGREIPSPNG

RAIRGAIQTDAAINAGNSGGPLIDSHGHVVGVNTATFTRKGTGASSGVNFAIPIDTVIRNVPNLIVYGTP

YSNRF

>Ca5_Ca20678_TIFY_Subfamily

MALLRMVQSHSHSQHNNNNNDNGGTHHLLNPVLHDFLGMKPSDTSADVRLPETSVSASSAGARGPFSTTS

ETVSEKQVGNHLEGVPFYGPRSDFTGTEISNRLVGNKRSNSDSSFMGSSRDAFQMVPDSFQNSHLMKVLR

NAFGGERSRRPNDDEVLHAMQSMKPSSSSQIFHPPSTTKLDANKWERSILTNIGPSMQHPPRGGQLMTPL

AHQLASNKIRDTNAGPSFISQSAADEGSRTGIKGPGVLSSINTSATATDKISSAVLLGGSRPKPLTNIVE

SFTPPSQHGLTSASRQMTIFYGGQAHVFDDVHPHKADVIMALAGSNGGSWSTAFSPKTTAKLTNESNLHS

GENETGLVSNVAFTQEHHGKLSVTGSSSRAVGGLGDRVSTPAGVHQGSIFAKDTRNPVQAADPSSEDKRA

L

>Ca08916-Ca1_TIFY10A-like

MSTSSEHSEISGQKPARSPEKSTFSQTCSLLSQYIKEKGSFKDLSLGMTCNTDPIGSPETSSHSATTMNF

FPNKENNLTPKNLTTMDLLTPQAALNNSNAIKGPKAAQLTMFYGGQVIVFDDFPADRAHELMSFASKGIS

QSHNNSVFTYTQSQPSFPINLVRTSADSTTPIVPSVNIVANTGAGSVLEHPQVPSRPIVCGNFLKPLTLK

FRMTHAHTHTYTYFKHMDYVVLIVNFLFCADLPIARKASLHRFLEKRKDRIAAKAPYQRTNTMEHVNKAT

ESMSWLGLGAKPTQI

>Ca09015-Ca1_TIFY3B-like

MDGVTVKVEPEQFMVLESSSIAADCVDGVSSNMGDVSMMNLSGNKSMPSSGLNAVIPNTSQLTIFYNGSI

CIYDGIPAEKVHEIMLIAAASAKSTEMKKIGKQSPILSTVPTRPSFPHGTIDNIASPQALCFPAKNSSIC

KLQEFPIARRHSLQRFLEKRRDRLGSKAPYPSSPSTKVADNLENNLCADNSPDSVSLKRPDEKFQPTISA

S

>Ca13423-Ca4_TIFY6A

MERDFLGLCSKESLSLPKEQFNNEGCKDSGILFLMYRITKLIKCSLVSQWDYQNHGGLNPIVKQNMRHVH

VWIGGFTQVSAVKWPFWNKVSAHSYLMPFNVSEEEKRATAGGLQKSFKHDGQGGIHYSLNPYPVQHNVSY

ANRPHDVKMFSVSVGSPFLKNHFATVGQNMNGANVMQPLFGGLLPVTAPHSVLPIVGTVTSSAEPCVKPS

APAPQLTIFYGGTVNVFNDITPETAQAIMLLAGNGVSASLDGAQPEVQAPISKFASGDDVPMSPPADIPP

CSGISSPLSVSSHTGPPFGSGASSSDEFLDAKPSKGPTPTISVSKVETPKIVNATTMFPSAIPQARKASL

ARFLEKRKERVMSAAPYNLNKKSEDAPTPNSMVANVSATTGTKTPSAKQG

>Ca01899-Ca6_TIFY5A-like

MRKKLNLELCLFPSSNSDLRCPTKEANESEVKDEQQHRPLTIVYDGKVCVCDATEIQAKSILMLANKEME

ERVRTPRSEPSSPTLLVSSHSHYHNHLYTPAGTSLSMKKSLQRFLQKRKNRIQEASPYHLKLNDQN

>Ca03206-Ca6_TIFY6B-like

MERDFMGLNSKEPLSFVKEEINDVGCKNSSFMKGSAAQWPFYNKVSNPTHLMSFKVSQDDNKTKILSDPI

TSAAGFMSVLSPTAFDSSHKRSAAEHQKCLNHDGQGGFHFSLTPYPIQHDVKMFSVSNNHFVTTGQNMNG

YNVTLPLLGGIPVTLPQSSLPTVGAVAGITESCNVKPTGPSSRLTIFYAGTVNVFDDISAEKAQAIMLLA

GNGLSMASNIAQTKVAAPGSKLAAGDGVPVSQHANTPPSSGHPSPISISSHAGTQSGCGSTSNDEFLAVK

TTGVLATLVSNLELPKVVNAATMLASEAIFYAAVPQARKASLARFLEKRKERVMNAAPYNFNKKSEECAA

A

>Ca16812-Ca7_TIFY11B

LLVVLLKMSTFPNTVSDSQRSGKAPEKFKFSQTCSLLSQFLKEKRISGDSTPGLFGKIKPKASTKDLLGN

KQNSDGGLRLNASAMDSLPQLVENPCIKKSNIRSTNSETPQLTIFYAGKMLVFDAFRPEKATEIMELATK

LASENSSREENPTSAPITSEKLKDSKVPQPKTALETPRENQVIGSDMRYPRRASLLKFLEKRKERVISRG

PYQINNHKIEGSSSGGEPKEQCSKHFDLNI

>Ca18389-Ca7_TIFY10A-like

MSSSSENSGFSGHKPAKLPEKSNFSHTCNLLSQYIKENGSFGGLTLGKPCTVETNGSPETSCNSGTTMEL

FPTNMTPQTQNLKTLNLLSPNDVPALENSSVFKEPKTAQLTMFYGGKIIVLDEFPANKVEELISFARTTK

WSTYASYNQTQPSVIPNLFPQAPSRLIVCEQPIARKASLHRFLEKRKDRIAAKAPYQKSNPISAPVKPVK

SIPWLGLGATSTQV

>Ca18427-Ca7_TIFY3B

MASADSINNNVGQRSDNINSFRHQDSVNNFSVNWPMAVTGQNAVTPQFAMLYNGSMPMAVTGQNAVTPQF

AMLYNGSMCVYDGIPAQKVHEIMMMASANAKSSEMKSGIPFTSLISTSPSSPQGGTSNNLASLQSVSFPV

EKSSICRIQEFPITRRQSLQMFLEKRKIRLGSKAPYTSSTSKKVNNVENNFSSVLRLLEGK

>Ca00368-Ca1_TIFY10A-like

MNHHHNLTPINFLSHQLPYHPPLFLQPMIPNSANFSVMKDSINKEAICEQMTIFYDGKVFVFDDIPAEKA

KEIMSFSTKGIISQNQNNYTHTFVHDHPQVPSIPIIYDLPMTRKASLHRFLEKRKDRIAGRAPYQTSKSA

TLNKPIDESMAWLSLAPQSPQDNKSECSSSSVLF

>Ca01249-Ca8_TIFY6B

LHLSKIYSFYNLEYKLICGCISDFFHHMEREFLGLNSKNIAWFNMKGDASNKPKDPVRSSGMQWSFSNKA

STVPQFLSFKNNTHEDRSRNTTMDPLASSGYMTISTKDAFDSNQKSFLAVTQESLSIGKQVTNKLGMTIY

PMQCSDHAQSICNQETRIFSVSNQSNQMCSVLQSNHATNGINMVNSVIKSQTLGSKSSATPLSVLPSIGS

LIGSTDLRNRNCSKSNGTPTQLTIFYGGSICVYDDISPQKAQAIMLLAGNGPKLQPEISVPSEKDGFIIS

QSYPSPLPHANSQPRGGSSSNNEHSIIRPIVPSIVPINHLESSIVATSLRSTPTKVIQPGLPQARKASLT

RSEKKGLPQARKASLTRFLEKRKERAMSTSPYYMCKKSSECNTLGSDSTSFSIDFSASSPQLATNLPLRT

TCMEV

>Ca11869-Ca4_TIFY4B

MNGGSTVPFRSILDKPLTQLTEDDISQLTREDCRRFLKEKGMRRPSWNKSQAIQQVISLKALLEPTDDDS

PAPVSSAIHHHHHHQPPQGNLNESPAKGTDPEDTGFRAAEDLQKSTSSAAEEPTDTNDANVVSPAGGCAP

SGSFGQMTSFYCGKVNVYDGVSPDKARSIMQLAASPSLFPQDNPSNKNAAVWASPCNLPIDKDGLFPTDT

ILQVVQTDKMVEHPLQYREKGSTARDADVEGLASRKVSLQRYLEKRKDRGRPKGKKLTGITSSNFEMYLN

LPVKVHASNGNSSRSSTDSPPQPRLPPVSSGSADNQQKVALPIDLNDKDVQEC

>Ca2-Ca29422_ZML-subfamily

MEPSIYGHSHPLNITAESDDGSGPDNTIEGHHHIQYETHGLNDGGVGGGVVVDEGTSDAVYGHGGGNSEL

ALQSFDDSSQLTLSFRGQVYVFDSVTPDKARFSIVQSVLLLLGGCELPQAGTTCVDAVPQQSQRGSTEFP

TKCSLPQRAASLIRFRQKRKERCFEKKVRYGVRQEVALRMHRNKGQFTSSKKQDGANSCGTDQDSGQDDS

QSVTFCTHCGISSKSTPMMRRGPSGPRSLCNACGLFWANRGALRDLSKRHPELSIVPAEHVDQGNGSDCG

TAIIPAHNNLAAFSENDNQALVADR

>Ca5-Ca21020_ZML-subfamily

LMAEQPLQFEDPAIDVVDDEDDDDDGGDDTMDELEETNVNSVNVAAASANQDDVVLPTTRASELTLSFEG

EVYVFPSVTHQKVQAVLLLLEGRDGQAGMPTVELPFDQSNKGMGSITKRSNLSRRIASLVRFREKRKERC

FDKKIRYTVRKEVAERMHRKKGQFASLKQNPSSSNCDSPQSVGQDGTPNPGSLRRCQHCGVNENNTPAMR

RGPAGPRTLCNACGLMWANKGTLRDLSKGGRNLSVEQSGLDPRIDVKPTVLEGELSVIQNEQGFSKIPSE

AIAAEGSNNHTLDPCDEELPESAEHLTNTLPLEIDQSSDNDDEQEPLAELSNPSDTGMSNPSDTDVDIPG

NFD

>Ca5_Ca21022-ZML-subfamily

MYNSMNMSDQIKNNEDADNHIHYDSHTLEDGSGEGEGPIHDVSLEDVYVSGDGNHADMSIQRFDDSSQLT

LSFRGQVYVFDSVTPEKVQSVLLLLGGCELSSGSQCLDTSPLNQRSGTEFPTRCSQPQRAASLIRFRQKR

KERNFDKKVRYGVRQEVALRMQRSKGQFTSSKKQDGANNWGSDPESGQDVVQSETSCTHCGTSSKSTPMM

RRGPSGPRTLCNACGLFWANRGTLRDLSTSKRHHEQHTLAAPPEQGTLRDLSNSKRNHELHTLASPKTVD

EGNDLNCRTALPAPNDTVDDKTT

>Ca6_Ca05650-ZML-subfamily

MDGIRGGDSRMQISDGQHPVHVPYEHHGLHHMSNGNGMDDDHNNGRDSNCGGSESVEGDIPSNHGNLHDN

HNLMMDQGNDIGDQLTLSFQGQVYVFDSVSPEKVQSVLLLLGGREMHPTLPSVPISPDENNRGFIGTPQK

FSVPQRLASLNRFREKRKERNFDKKIRYTVRKEVALRMQRNKGQFTSSKSNHDESASAAMNGGTNEGLIA

DNNGSQQHDIVCRHCGISEKCTPMMRRGPEGPRTLCNACGLMWANKGALRDLSRAATLPVAHNSPLNKNE

NKNSETNQIVLRDAAESSS

>Ca6-Ca02171_ZML-subfamily

MDTLNSCENELMVVPMHCTNGASVSDEGGEESQPIATTSRASELTISFEGEVYVFPSVTPQKVQAVLLLL

GRQETPDSTPTSNFLVQQNCQDIWGINDPSRSSKLSRRIASLVRFREKRKERCFEKKIRYTCRKEVAQRM

HRKNGQFASLKEDYISPAENQDSSNGTPCPESTERRCQHCGIGEKSTPAMRRGPAGPRSLCNACGLMWAN

KGTLRDLSKAGRIAFEQNELDTSTDIKPSTTEPGNSCTVQDKEGSPQENKPVPIDVKQSPETENEQYILE

SAEAVTDNLSIQLENNVLDLHEQDNTMEDFADASGTEFEIPAGFDDQVDIDDSNMRTYWL

**Protein sequence of chickpea NINJA**

>Ca01446-CaNINJA

MEDESGIELSLGLSCGGSSTKPKSKNGSSSDTRAEEAGRGGKMVDDFKSMFNTDPQKPESIAGTRRSDSS

KPEENFFSDLSKVKEDNASLNLNGRGYLVANNNNNNKPIEIEENKRLEVVNKRRMSFDDIRNQKKHDSDV

HHVDMHDRARTSHISLTEDGSTAENEDVADSEADNSTSRPLSHHSDGSKGFIRVGGASSDAPKEVRGVAD

SSANGQKRYTPSTEKDFKHANMNYSGASFSAQQVNMMMGVPYSTVKESNLVGGPNPQMPGVMHVMPTSTG

ERAGAQSVSNGSLPMMFGYSSVQLPMLDKDSSWGLASRPQQLHPSFAGRGPTNSASAALHLNNISEAMPY

EGRPLERTKGDGKQRAAEESSFSQPEDMKGSSTNLRAKDVSEHSKGEGSTIDFSNIKPGLAADVKFGGCG

SYPNLPWVSTTGSNGRTISGVTYRYSTNQIRIVCACHGSHMTPEDFVRHANDDQANSDGNAVLGTVANGN

PGASSHS

**Protein sequence of Ca4_TIFY4B orthologues**

>AT4G14713.1_PPD1_TIFY4A_PEAPOD1[Arabidopsis]

MDVGVSPAKSILAKPLKLLTEEDISQLTREDCRKFLKDKGMRRPSWNKSQAIQQVLSLKALYEPGDDSGA

GIFRKILVSQPVNPPRVTTTLIEPSNELEACGRVSYPEDNGACHRMDSPRSAEFSGGSGHFVSEKDGHKT

TISPRSPAETSELVGQMTIFYSGKVNVYDGIPPEKARSIMHFAANPIDLPENGIFASSRMISKLISKEKM

MELPQKGLEKANSSRDSGMEGQANRKVSLQRYREKRKDRKFSKAKKCPGVASSSLEMFLNCQPRMKAAYS

QNLGCTGSPLHSQSPESQTKSPNLSVDLNSEGI

>AT4G14720.1_PPD2_TIFY4B_PEAPOD2[Arabidopsis]

MDVGVTTAKSILEKPLKLLTEEDISQLTREDCRKFLKEKGMRRPSWNKSQAIQQVLSLKALYEPGDDSGA

GILRKILVSQPPNPPRVTTTLIEPRNELEACGRIPLQEDDGACHRRDSPRSAEFSGSSGQFVADKDSHKT

VSVSPRSPAETNAVVGQMTIFYSGKVNVYDGVPPEKARSIMHFAANPIDLPENGIFASSRMISKPMSKEK

MVELPQYGLEKAPASRDSDVEGQANRKVSLQRYLEKRKDRRFSKTKKAPGVASSSLEMFLNRQPRMNAAY

SQNLSGTGHCESPENQTKSPNISVDLNSDLNSEDN

>BBD71115.1_VmPPD[Vigna-mungo]

MNVGGTATFRSILDKPLNQLTEDDISQLTREDCRRFLKEKGMRRPSWNKSQAIQQVISLKALLEPSDDDS

PPPPTPMHHQPHAPQPQANLTQPPPKAPPPEEPAFLAADDIQKSASSGEKPTETNDTNTNANVASPRGCA

TSGSIGQMTIFYCGKVNVYDGVSPDKARAIMQLAASPVQFTQDDPLHGNASAWSSPCHLPMDKDVLIPVD

TAILQVAQADKMVEYPLQYREKGSITRDADIDGQASRKVSLQRYREKRKDRGRFKGKKLTGITSSNFEMY

LNLPVKVHASNGNSSRSSTSSPPQPRLPLASSVSADNQLKVGLPIDLNDKDVQEC

>AKN91664.1_BIG-SEEDS2[GLYMA]

MNGGATTATFRSILDKPLNQLTEDDISQLTREDCRRFLKEKGMRRPSWNKSQAIQQVISLKALLEPSDDD

TPPPTAMHHRSHAPPPPPQPQSQVNLTEPPPPPKAPPPEESSFHAAEDIQKPASSGEKPSETNDTNTNVA

SPKGCATSGSFGQMTIFYCGKVNVYDGVSPDKARAIMQLAVSPVQFTQDDPSNGNAAVWPSPCHLPMDKD

VLIPVDTTILQVAQSDKMMEYPLQYREKGSIARDADVEGQASRKVSLQRYLEKRKDRGRLKGKKLTGITS

SNFEMYLNLPVKVHASNGNSSRSSTSSPPQPRLPLVSSGSADNQLKVALPIDLNDKVSLQMFKNAKTLTR

>AKN91689.1_BIG-SEEDS1[GLYMA]

MNGGATTATFRSILDKPLNQLTEDDISQLTREDCRRFLKEKGMRRPSWNKSQAIQQVISLKALLEPSDDD

TPPPPPPAMHHRSHAQPQPQVNLSEPPPPPPKAPPPEEPAFHAAEDIQKSASSGEKPTETNDTNTNVASP

KGCATSGSFGQMTIFYCGKVNVYDRVSPDKARAIMQLATSPVQLTQDDPLNGNAAVWTSPCHLPMDKDVL

VPVDTTILQVAQADKMVEYPLQYREKGSIARDADVEGQEHRKVSLQRYLEKRKDRGRLKGKKLTGITSSN

FEMYLNLPVKVHSSNGNSSRSSTSSPPQPRLPLVSSGSDQLKVALPIDLNDKVSLQMFKNAKIQTR

>AKN91692.1_BIG-SEEDS1[M.Truncatula]

MNGGSTVSFRSILDRPLNQLTEDDISQLTREDCRRFLKDKGMRRPSWNKSQAIQQVISLKALLEPTDDDI

PATVGVGVSSAIHHHHHHHPPQPPPKALDPEDTALELQKSTSPVAERPTETNDANVVNNPGGCAPSGSFG

QMTIFYCGKVNVYDGVSPDKARSIMQLAAACPSSFPQDNPSNKNAAVWASPCNLPIDKEVLFPTDTAILQ

VAQTDKMVEYPLQYREKGSTARDADVEGQASRKVSLQRYLEKRKDRGRSKGKKLTGITSSNFEMYLNLPV

KLHASNGNSSRSSTDSPPQPRLPLVSSGSAENQPKVTLPIDLNDKDVQEC

>AXL93707.1_ELE1_TIFY_4B[L.japonicus]

MNAGATVIRSILDKPLNQLTEDDISQLTREDCRRFLKDKGMRRPSWNKSQAIQQVISLKALLEPTDDDSP

APVSSAIHRHHHHHPQPPQGNLSEAPVKSPNREIGVRAVEDVQKSISPAAEKPTETNDAPAGGCAPSGSF

GQMTIFYCGKVNVYDGVSPDKAQSIMQLAASPSPFPQDNPLNKNAAVWASSCNIPIDKDVLFPTNTAILQ

VSQTDKMVEYPLQYREKGSTPRDADVEGQASRKVSLQRYLEKRKDRGRPKGKKLTGITSSNFEMYLNLPV

KVHASNGNSSRSSTDSPPQTRLPLVSSGSADNQPKVALPIDLNDKDVQEC

>gnl.PsELE1[Pisum-sativum]

MNAGATVIRSILDKPLNQLTEDDISQLTREDCRRFLKDKGMRRPSWNKSQAIQQVISLKALLEPTDDDSP

APVSSAIHRHHHHHPQPPQGNLSEAPVKSPNREIGVRAVEDVQKSISPAAEKPTETNDAPAGGCAPSGSF

GQMTIFYCGKVNVYDGVSPDKAQSIMQLAASPSPFPQDNPLNKNAAVWASSCNIPIDKDVLFPTNTAILQ

VSQTDKMVEYPLQYREKGSTPRDADVEGQASRKVSLQRYLEKRKDRGRPKGKKLTGITSSNFEMYLNLPV

KVHASNGNSSRSSTDSPPQTRLPLVSSGSADNQPKVALPIDLNDKDVQEC
