# Supplementary material for: Fine Mapping of a Vigor QTL in Chickpea (Cicer arietinum L.) Reveals a Potential Role for Ca4_TIFY4B in Regulating Leaf and Seed Size
Source: Front Plant Sci. 2022 Feb 24;13:829566. doi: 10.3389/fpls.2022.829566 (PMC8908238; doi:10.3389/fpls.2022.829566)
Supplement: Supplementary File 1 — Primer sequences. [file Data_Sheet_1.zip › Supplementary Material/Supplementary File S5. Rupali and Genesis836 Ca4_TIFY4B and CaNINJA sequences..DOCX]

**Supplementary file 5. cDNA sequences for *CaNINJA* (Ca01446) and *Ca4_TIFY4B* (Ca11869) from Rupali and Genesis836** **after cloning into the vector pENTR™/D-TOPO®.** No polymorphism was detected between Rupali and Genesis836 in *CaNINJA*. For *Ca4_TIFY4B*, a SNP (in red) was confirmed between Rupali (*CaTIFY4B_R*) and Genesis836 (*CaTIFY4B_G*). Sequence IDs are from *CDC Frontier reference genome v2.6.3;* <http://doi.org/10.7946/P2G596>, Edwards, 2016; Ruperao, 2016)

**CaNINJA** (Ca01446)

ATGGAGGACGAGAGCGGGATTGAGCTCAGTTTGGGTTTATCTTGTGGTGGTTCATCAACCAAACCCAAGAGTAAGAATGGAAGCTCCTCGGATACTAGGGCAGAAGAAGCCGGTAGAGGTGGCAAGATGGTGGATGATTTCAAAAGCATGTTTAATACTGATCCTCAGAAGCCAGAATCAATTGCTGGTACTCGAAGGAGCGATTCCTCGAAACCTGAAGAGAACTTCTTTAGTGACCTTTCAAAGGTCAAAGAAGATAATGCTTCTTTGAATTTAAACGGGAGAGGATATCTGGTTGCAAACAACAATAACAACAATAAACCTATTGAAATTGAGGAAAATAAACGGTTAGAGGTAGTAAATAAGCGAAGAATGTCTTTTGATGACATACGTAATCAAAAGAAGCACGACAGCGATGTTCATCATGTTGATATGCATGACAGGGCAAGAACATCTCATATTTCTTTAACAGAAGATGGCTCAACTGCAGAAAATGAAGATGTTGCTGATTCTGAAGCTGATAACTCTACCTCGAGGCCTCTCTCACACCATAGTGATGGTTCCAAAGGATTCATCAGAGTCGGTGGTGCTTCTTCTGATGCTCCCAAAGAGGTACGCGGAGTCGCTGACTCAAGCGCCAACGGGCAGAAGAGATATACTCCATCCACCGAAAAAGATTTTAAACATGCAAACATGAATTATAGTGGTGCTTCCTTCTCTGCTCAACAAGTAAATATGATGATGGGTGTACCTTACTCTACAGTAAAAGAGTCCAACTTGGTTGGTGGACCGAACCCTCAAATGCCTGGAGTGATGCACGTGATGCCTACTTCCACCGGTGAACGTGCGGGAGCTCAATCTGTGAGTAATGGAAGCTTGCCAATGATGTTTGGATATTCTTCTGTTCAGCTTCCCATGTTGGATAAGGATAGCTCATGGGGATTGGCTTCTCGTCCACAACAGTTACATCCTTCCTTTGCTGGGAGAGGTCCAACTAACTCAGCTTCAGCTGCGTTACACCTAAACAATATATCCGAGGCCATGCCATATGAAGGAAGGCCACTAGAACGAACCAAAGGTGACGGAAAACAGCGTGCCGCTGAAGAAAGCTCATTCTCACAACCTGAAGATATGAAAGGAAGCAGCACAAACCTCAGGGCCAAAGATGTATCCGAACATTCGAAAGGAGAAGGTTCGACCATTGACTTTTCAAATATTAAGCCGGGACTCGCTGCAGATGTGAAATTTGGAGGATGCGGTTCATACCCAAATCTACCTTGGGTATCCACCACAGGCTCAAATGGAAGAACAATATCAGGTGTTACTTACAGGTACAGCACTAACCAAATCAGAATTGTTTGTGCATGTCATGGCTCTCACATGACCCCCGAGGATTTCGTTCGCCATGCAAACGACGATCAAGCCAATTCAGATGGCAATGCAGTTTTGGGAACCGTAGCAAACGGAAATCCAGGTGCCTCTTCTCACAGTTAG

**CaTIFY4B_G (**Ca11869)

ATGAACGGCGGAAGCACCGTTCCCTTCCGATCCATCCTCGACAAACCCCTTACCCAGCTCACCGAAGATGACATTTCTCAACTCACACGCGAAGACTGTCGCAGATTCCTCAAAGAAAAAGGGATGCGCAGGCCTTCCTGGAACAAATCACAAGCGATCCAGCAAGTTATTTCTCTCAAAGCACTTCTCGAACCTACCGACGACGATTCTCCCGCCCCCGTCTCCTCCGCCATACACCACCACCACCACCATCAACCTCCACAAGGGAATTTGAATGAATCTCCGGCGAAAGGAACGGATCCTGAAGATACTGGTTTTCGTGCTGCGGAGGATCTTCAGAAATCTACTTCATCTGCTGCGGAAGAACCTACGGACACTAATGATGCCAACGTTGTTAGCCCCGCCGGAGGGTGCGCACCTAGCGGATCATTTGGGCAAATGACAATTTTCTACTGTGGTAAGGTTAATGTCTATGATGGAGTCTCACCGGATAAGGCACGATCAATCATGCAGCTAGCTGCAAGTCCGTCCCTGTTTCCTCAGGATAATCCTTCAAATAAAAATGCTGCAGTTTGGGCTTCTCCTTGCAACTTACCGATTGATAAGGATGGTCTCTTCCCCACTGACACAATCCTTCAAGTTGTTCAAACAGATAAGATGGTGGAACATCCTCTGCAATACAGGGAGAAAGGGAGCACAGCTCGTGATGCTGATGTAGAGGGTCTGGCAAGCAGAAAAGTGTCACTGCAGCGATATCTTGAAAAGCGAAAGGACAGGGGAAGACCAAAAGGAAAGAAACTGACTGGCATAACTTCATCTAACTTTGAGATGTATTTGAACCTTCCAGTGAAGGTCCATGCCTCAAATGGGAATTCAAGTCGTAGTAGCACTGACTCTCCTCCACAGCCTAGACTGCCTCCAGTTTCCAGTGGCTCAGCTGACAACCAGCAAAAAGTTGCCCTTCCCATTGATCTCAATGATAAAGATGTTCAAGAATGCTAA

**CaTIFY4B_R (**Ca11869)

ATGAACGGCGGAAGCACCGTTCCCTTCCGATCCATCCTCGACAAACCCCTTACCCAGCTCACCGAAGATGACATTTCTCAACTCACACGCGAAGACTGTCGCAGATTCCTCAAAGAAAAAGGGATGCGCAGGCCTTCCTGGAACAAATCACAAGCGATCCAGCAAGTTATTTCTCTCAAAGCACTTCTCGAACCTACCGACGACGATTCTCCCGCCCCCGTCTCCTCCGCCATACACCACCACCACCACCATCAACCTCCACAAGGGAATTTGAATGAATCTCCGGCGAAAGGAACGGATCCTGAAGATACTGGTTTTCGTGCTGCGGAGGATCTTCAGAAATCTACTTCATCTGCTGCGGAAGAACCTACGGACACTAATGATGCCAACGTTGTTAGCCCCGCCGGAGGGTGCGCACCTAGCGGATCATTTGGGCAAATGACAAGTTTCTACTGTGGTAAGGTTAATGTCTATGATGGAGTCTCACCGGATAAGGCACGATCAATCATGCAGCTAGCTGCAAGTCCGTCCCTGTTTCCTCAGGATAATCCTTCAAATAAAAATGCTGCAGTTTGGGCTTCTCCTTGCAACTTACCGATTGATAAGGATGGTCTCTTCCCCACTGACACAATCCTTCAAGTTGTTCAAACAGATAAGATGGTGGAACATCCTCTGCAATACAGGGAGAAAGGGAGCACAGCTCGTGATGCTGATGTAGAGGGTCTGGCAAGCAGAAAAGTGTCACTGCAGCGATATCTTGAAAAGCGAAAGGACAGGGGAAGACCAAAAGGAAAGAAACTGACTGGCATAACTTCATCTAACTTTGAGATGTATTTGAACCTTCCAGTGAAGGTCCATGCCTCAAATGGGAATTCAAGTCGTAGTAGCACTGACTCTCCTCCACAGCCTAGACTGCCTCCAGTTTCCAGTGGCTCAGCTGACAACCAGCAAAAAGTTGCCCTTCCCATTGATCTCAATGATAAAGATGTTCAAGAATGCTAA
