# Supplementary material for: Fine Mapping of a Vigor QTL in Chickpea (Cicer arietinum L.) Reveals a Potential Role for Ca4_TIFY4B in Regulating Leaf and Seed Size
Source: Front Plant Sci. 2022 Feb 24;13:829566. doi: 10.3389/fpls.2022.829566 (PMC8908238; doi:10.3389/fpls.2022.829566)
Supplement: Supplementary File 1 — Primer sequences. [file Data_Sheet_1.zip › Supplementary Material/Supplementary Tables S1 - S3.pdf]

Supplementary Table S1. Interactions tested in Y2H assay. (A) NINJA as bait, TIFY4B_R and TIFY4B_G as preys. **(B)** TIFY4B_R and TIFY4B_G as baits, NINJA as prey. pDEST™32 is the destination vector for bait construction, containing DNA binding domain GAL4-DBD; pDEST™22 is the destination vector for prey construction containing activation domain GAL4-AD.

(A)

No	LEU2 Plasmid (Bait)	TRP1 Plasmid (Prey)	Purpose	Interaction ID
1	pDEST™32	pDEST™22/TIFY4B_G	Negative activation control	GAL4-DBD-TIFY4B_G
2	pDEST™32/NINJA	pDEST™22/TIFY4B_G	Experimental interaction	GAL4-NINJA-TIFY4B_G
3	pDEST™32/NINJA	pDEST™22/TIFY4B_R	Experimental interaction	GAL4-NINJA-TIFY4B_R
4	pDEST™32	pDEST™22/TIFY4B_R	Negative activation control	GAL4-DBD-TIFY4B_R
5	pEXP™32/RalGDS-wt	pEXP™22/Krev1	Strong positive interaction control	GAL4-Krev1-RalGDS
6	pDEST™32/NINJA	pDEST™22	Negative activation control	GAL4-NINJA-AD

(B)

No	LEU2 Plasmid (Bait)	TRP1 Plasmid (Prey)	Purpose	Interaction ID
1	pDEST™32	pDEST™22/NINJA	Negative activation control	GAL4-DBD-NINJA
2	pDEST™32/TIFY4B_G	pDEST™22/NINJA	Experimental interaction	GAL4-TIFY4B_G-NINJA
3	pDEST™32/TIFY4B_R	pDEST™22/NINJA	Experimental interaction	GAL4-TIFY4B_R-NINJA
4	pDEST™32/TIFY4B_G	pDEST™22	Negative activation control	GAL4-TIFY4B_G-AD
5	pDEST™32/TIFY4B_R	pDEST™22	Negative activation control	GAL4-TIFY4B_R-AD
6	pEXP™32/Krev1	pEXP™22/RalGDS-wt	Strong positive interaction control	GAL4-Krev1-RalGDS

Supplementary Table S2. F_2 HIF phenotyping in greenhouse conditions. HIF = Heterogeneous Inbred Family, 100SDW = Average weight of 100 seeds,. Values are the mean (± standard error mean) of measurements. Bold text indicates HIFs for which all four traits were significantly different ($P < 0.05$) between progeny carrying a Rupali (R) or Genesis836 (G) allele within the recombinant *Ca4_Vqtl* interval.

HIF	Allele	Number of progeny	Leaf size at 13 weeks (cm2)	100SDW (g)	Seed size (mm^2)	Seed Number
**50.22.94**	**G**	**10**	**35.6 ± 1.4**	**18.7 ± 1**	**43.8 ± 1.3**	**43 ± 7**
	**R**	**8**	**46.8 ± 2.5**	**26.9 ± 0.5**	**52.9 ± 0.7**	**25 ± 4**
**50.13.189**	**G**	**13**	**28.2 ± 1**	**19.6 ± 0.3**	**40.4 ± 0.4**	**56 ± 3**
	**R**	**14**	**41.2 ± 1.6**	**26.1 ± 0.3**	**50.6 ± 0.4**	**26 ± 3**
**50.13.10**	**G**	**16**	**25.6 ± 0.7**	**20.1 ± 0.3**	**40.1 ± 0.4**	**50 ± 4**
	**R**	**11**	**35.1 ± 1.4**	**28.4 ± 0.8**	**51.6 ± 1**	**26 ± 5**
**50.4.172**	**G**	**13**	**31.1 ± 1**	**19.8 ± 0.5**	**42.5 ± 0.6**	**50 ± 4**
	**R**	**8**	**40.9 ± 1.6**	**25.3 ± 0.4**	**51 ± 0.6**	**37 ± 4**
**50.23.252**	**G**	**14**	**30 ± 1**	**20.5 ± 0.2**	**37.7 ± 0.3**	**34 ± 4**
	**R**	**12**	**43.7 ± 1.5**	**26.7 ± 0.6**	**51.6 ± 0.7**	**`26 ± 3**
**50.23.303**	**G**	**18**	**32 ± 1**	**19.7 ± 0.2**	**44.7 ± 0.3**	**53 ± 5**
	**R**	**9**	**41.3 ± 1.7**	**26 ± 0.4**	**50.4 ± 0.6**	**31 ± 3**
**50.7.81**	**G**	**12**	**35.8 ± 1**	**19 ± 0.3**	**36.6 ± 0.4**	**45 ± 3**
	**R**	**16**	**49.2 ± 1**	**25.7 ± 0.4**	**43.7 ± 0.5**	**27 ± 2**
**50.22.83**	**G**	**5**	**22.5 ± 1.3**	**19.5 ± 0.7**	**40.7 ± 0.9**	**61 ± 7**
	**R**	**6**	**32 ± 2**	**24.8 ± 1.7**	**48.1 ± 2.3**	**39 ± 2**
**50.13.190**	**G**	**5**	**22.5 ± 1.6**	**17.1 ± 0.6**	**37.3 ± 0.8**	**77 ± 8**
	**R**	**14**	**32.6 ± 1.1**	**25.8 ± 0.9**	**49.4 ± 1.2**	**30 ± 3**
50.4.436	G	6	34.1 ± 2.6	19.9 ± 0.4	41.4 ± 3.7	44 ± 8
	R	7	33.5 ± 1.7	19.4 ± 0.6	40.7 ± 0.8	41 ± 8
50.22.143	G	17	25.2 ± 0.8	19.6 ± 0.8	41.7 ± 1	42 ± 5
	R	16	25.1 ± 0.8	20.9 ± 0.5	42.8 ± 0.6	46 ± 5
50.7.100	G	8	26.5 ± 1.2	20 ± 0.5	41.9 ± 0.6	29 ± 7
	R	8	29.2 ± 1.6	20.5 ± 0.8	42.3 ± 1	33 ± 6
15.5.334	G	20	17 ± 0.6	17 ± 0.4	37.8 ± 0.5	49 ± 2
	R	12	16.4 ± 0.7	16.8 ± 0.3	36.6 ± 0.4	49 ± 3
**15.5.397**	**G**	**15**	**18.6 ± 0.7**	**16.3 ± 0.3**	**36.3 ± 0.4**	**42 ± 3**
	**R**	**16**	**26.2 ± 1.4**	**22.6 ± 0.7**	**45 ± 0.9**	**29 ± 3**
**15.4.147**	**G**	**15**	**26 ± 1.1**	**18.6 ± 0.7**	**38.9 ± 1.7**	**55 ± 4**
	**R**	**11**	**38.5 ± 0.9**	**24.2 ± 0.6**	**47.6 ± 0.8**	**44 ± 2**

Supplementary Table S3. List of genes inside the reduced *Ca4_Vqtl* interval. SNP-level sequence variation within each gene was identified using sequence capture data for Genesis836 and Rupali (Nguyen *et al*., 2021).

No	Gene ID (Ref V.2.6.3)	Start	End	SNP position	SNP location	Amino acid substitutions	Putative function
1	Ca11861	12878013	12875640				Emp24/gp25L/p24 family protein n=2 Tax=Papilionoideae RepID=A0A072VQS5_MEDTR
2	Ca11857	12881188	12888196	Ca4_12881281	Intron		Aldo/keto reductase family oxidoreductase n=3 Tax=Papilionoideae RepID=A0A072VPP5_MEDTR
3	Ca11870	12896582	12889862				Dyggve-melchior-clausen syndrome protein n=2 Tax=Papilionoideae RepID=A0A072W0G2_MEDTR
4	Ca11871	12904424	12905788	Ca4_12903671	Promoter		Heat shock transcription factor A3 n=1 Tax=Medicago truncatula RepID=A0A072VQ54_MEDTR
5	Ca11868	12912373	12908997				1,2-dihydroxy-3-keto-5-methylthiopentene dioxygenase n=2 Tax=Papilionoideae RepID=A0A072VQU1_MEDTR
6	Ca11867	12918838	12915888				PREDICTED: uncharacterized protein LOC101502642 n=1 Tax=Cicer arietinum RepID=UPI00032AC57B
7	Ca11865	12923202	12921407	Ca4_12922166	Intron		1,2-dihydroxy-3-keto-5-methylthiopentene dioxygenase n=3 Tax=Papilionoideae RepID=I3SWK2_MEDTR
8	Ca11869	12932150	12926475	Ca4_12929674	Exon	Ser/lle (non-conservative)	PREDICTED: protein TIFY 4B-like isoform X1 n=4 Tax=Cicer arietinum RepID=UPI00032A88EF
9	Ca11864	12951238	12951649				PREDICTED: EPIDERMAL PATTERNING FACTOR-like protein 5 n=1 Tax=Cicer arietinum RepID=UPI00032A60FE
10	Ca11866	12974745	12971733				PREDICTED: uncharacterized protein LOC101505664 isoform X1 n=2 Tax=Cicer arietinum RepID=UPI00032AA9D8
